# Charitable Institutions of Victoria

**Published:** 1904-07-09

**Authors:** 


### CHARITABLE INSTITUTIONS OF VICTORIA.

ALTHOUGH the report of the charitable institutions of Victoria for the year ending June 1903 has naturally been affected by the drought, the financial position of the institutions at the close of the financial year generally was sound. The Inspector of Charities states that the overdrafts, although amounting in all to £20,833, were spread over 78 institutions, and only six of these closed the year with a debit balance of over £1,000. Any charitable institution with a debit balance of less than £1,000 cannot, he considers, be financially embarrassed, as the indebtedness could easily be discharged by special local efforts. There have also in some quarters been praiseworthy and successful attempts towards economy. The Inspector's admonitions against spending money unnecessarily have had the result that many institutions have been able to show decreased expenditure, notably the Bendigo Hospital and the Children's Hospital. The cost of the former was £7,642 for the twelve months ending June 1902, and only £6,779 for that ending June 1903. The Children's Hospital spent £8,779 in 1901–1902, and only £8,107 in 1902–1903. The Inspector goes on to point out that it is not the actual cost of patients which embarrasses an institution's finances, but rather the upkeep of the institution, and that the closing of wards of which much has been heard of late years, is not productive of material saving, though a cruel hardship to the poor. He points out that the number of patients treated during the year in hospitals proper was 23,125 in-patients and 77,780 out-patients, and the number of nurses and attendants boarded at the institutions, 881. The total expenditure on hospitals proper was £125,326, the upkeep being £71,756, to which the board of the 881 nurses and attendants, £22,906, must be added. This makes a total of £94,662, leaving £30,664 only as the actual cost of the patients themselves, showing that the patients are actually responsible for about a quarter only of the total expenses of a hospital. Another unnecessary source of expenditure results from the idea that committees must periodically erect additional structures, whether required or not. In this way not only are amounts which should be utilised for maintenance placed to building account, but the additional building means in many cases an increased maintenance cost. A third case of extravagance is the amount paid for collection of subscriptions which frequently ranges as high as 42 per cent., a fact which is sufficient in itself to seriously prejudice donations for charitable purposes.

The Government grant for the hospitals 1903–1904 is fixed at £100,000, an increase of £10,000 over that allotted the previous year. It is considered that this should be amply sufficient for all requirements, and previous experience has shown that a high Government grant frequently leads to extravagance in management, whilst with a reduced grant economical tendencies prevail. The amount of municipal grant to charity during the year was only £10,783, and if these municipalities cannot be persuaded to contribute more liberally it may become a matter for the committees of hospitals in Victoria to consider whether they will not decline to any longer receive cases of an infectious nature, such as typhoid, diphtheria, etc. Such a refusal would force the municipal bodies themselves under the Health Act to provide suitable accommodation and treatment for the patients at much expense. There is also apparent a great need for more systematic collection of small sums. Very few sums under £1 are sent to the hospitals as voluntary contributions, and yet the Austin Hospital for Incurables, when the committee made a special appeal for small sums, in a few weeks received £700, mostly in sums of 1d. and 3d. Amongst gratifying records is the fact that £18,416 was contributed during the year by patients in the form of admission tickets, for which patients pay not necessarily a fixed sum, but what each one feels he can afford. The result is very satisfactory.

